# Orofacial manifestations in mucopolysaccharidoses: a comprehensive clinical and radiographic evaluation of 35 pediatric cases

**DOI:** 10.1186/s13005-025-00563-0

**Published:** 2025-12-22

**Authors:** Dilşah Çoğulu, Sema Kalkan Uçar, Ebru Canda, Elif Kantar Atila, Ali Rıza Alpöz, Mahmut Çoker

**Affiliations:** 1 Department of Pediatric Dentistry, Ege University Faculty of Dentistry, Bornova, İzmir, Turkey; 2 Department of Pediatrics, Division of Metabolism and Nutrition, Ege University Faculty of Medicine, İzmir, Turkey; 3 Private Dentist, İzmir, Turkey

**Keywords:** Mucopolysaccharidosis, Oro-dental finding, Pediatric dentistry

## Abstract

**Objective:**

Mucopolysaccharidoses (MPS) represent a heterogeneous group of rare, inherited lysosomal storage disorders characterized by the accumulation of glycosaminoglycans in various tissues, including the orofacial region. This study aims to investigate the orofacial features and radiographic manifestations in a cohort of pediatric patients diagnosed with MPS.

**Materials and methods:**

Thirty-five patients (18 males, 17 females) diagnosed with different subtypes of MPS underwent comprehensive oral and radiographic examinations. The presence and frequency of clinical and radiographic findings were documented. Radiographic analysis was feasible in 26 patients, with 9 excluded due to severe intellectual disability. The decayed, missing, and filled teeth (DMFT/dft); decayed, missing, and filled surfaces (DMFS/dfs) indices were recorded according to WHO criteria. The dental plaque scores were determined according to Silness & Löe plaque index. Data was analysed using SPSS 25.0, with descriptive statistics summarizing variables and Fisher’s Exact test applied for subgroup comparisons by MPS subtype; significance was set at *p* < 0.05.

**Results:**

The most frequent orofacial findings included tongue thrusting (80%, *n* = 28/35), limited mouth opening (71%, *n* = 25/35), macroglossia (71%, *n* = 25/35), and anterior open bite (54%, *n* = 19/35). Radiographic analysis revealed a thin mandibular cortex in 38% (*n* = 10/26) of cases, impacted teeth in 27% (*n* = 7/26), and a short mandibular ramus in 23% (*n* = 6/26). This is the first reported case of a talon cusp, a rare dental anomaly occurring in a patient with MPS-IV, highlighting a previously unrecognized association in the literature. The distribution of MPS types was: MPS-I (*n* = 3), MPS-II (*n* = 6), MPS-III (*n* = 8), MPS-IV (*n* = 10), MPS-VI (*n* = 7), and MPS-VII (*n* = 1).

**Conclusions:**

Orofacial manifestations are frequently observed in patients with MPS and may serve as early indicators of the disorder, particularly within dental settings. Recognition of these features is essential, as they underscore the need for multidisciplinary care and routine dental evaluations to mitigate potential complications and enhance overall quality of life.

**Clinical relevance:**

Orofacial abnormalities are common in children with MPS and may provide early clues to the diagnosis. This study emphasizes the importance of dental evaluations in identifying these signs, including the first reported case of a talon cusp in a patient with MPS. Early recognition by dental professionals can support timely diagnosis, guide appropriate referrals, and improve patient outcomes through coordinated, multidisciplinary care.

## Introduction

Mucopolysaccharidoses (MPS) are a clinically diverse group of rare, inherited, progressive lysosomal storage disorders. Each of these disorders results from a deficiency or malfunction of specific lysosomal enzymes required for the degradation of glycosaminoglycans (GAGs), which are also known as mucopolysaccharidosis [1–3]. The enzymatic defects result in the progressive intracellular accumulation of undegraded or partially degraded GAGs, primarily heparan sulphate, dermatan sulphate, keratan sulphate, and chondroitin sulphate within lysosomes in multiple organ systems. This accumulation has been demonstrated to disrupt normal cellular architecture and function, culminating in widespread somatic, skeletal, cardiovascular, respiratory and neurological impairment, often with life-limiting consequences [4, 5].

**Fig. 1:**
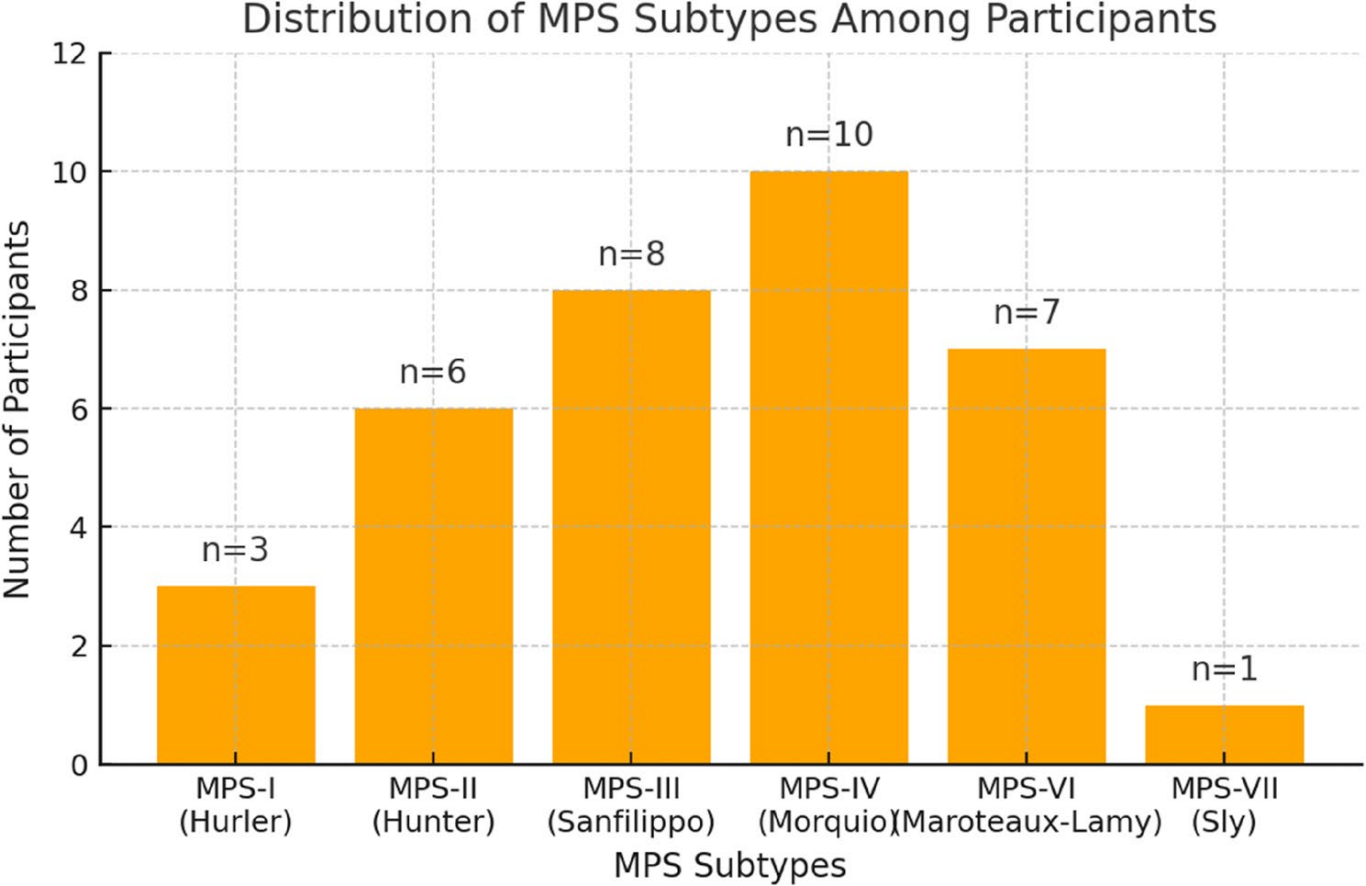
The distribution of MPS subtypes among participants (*n* = 35)

**Fig. 2:**
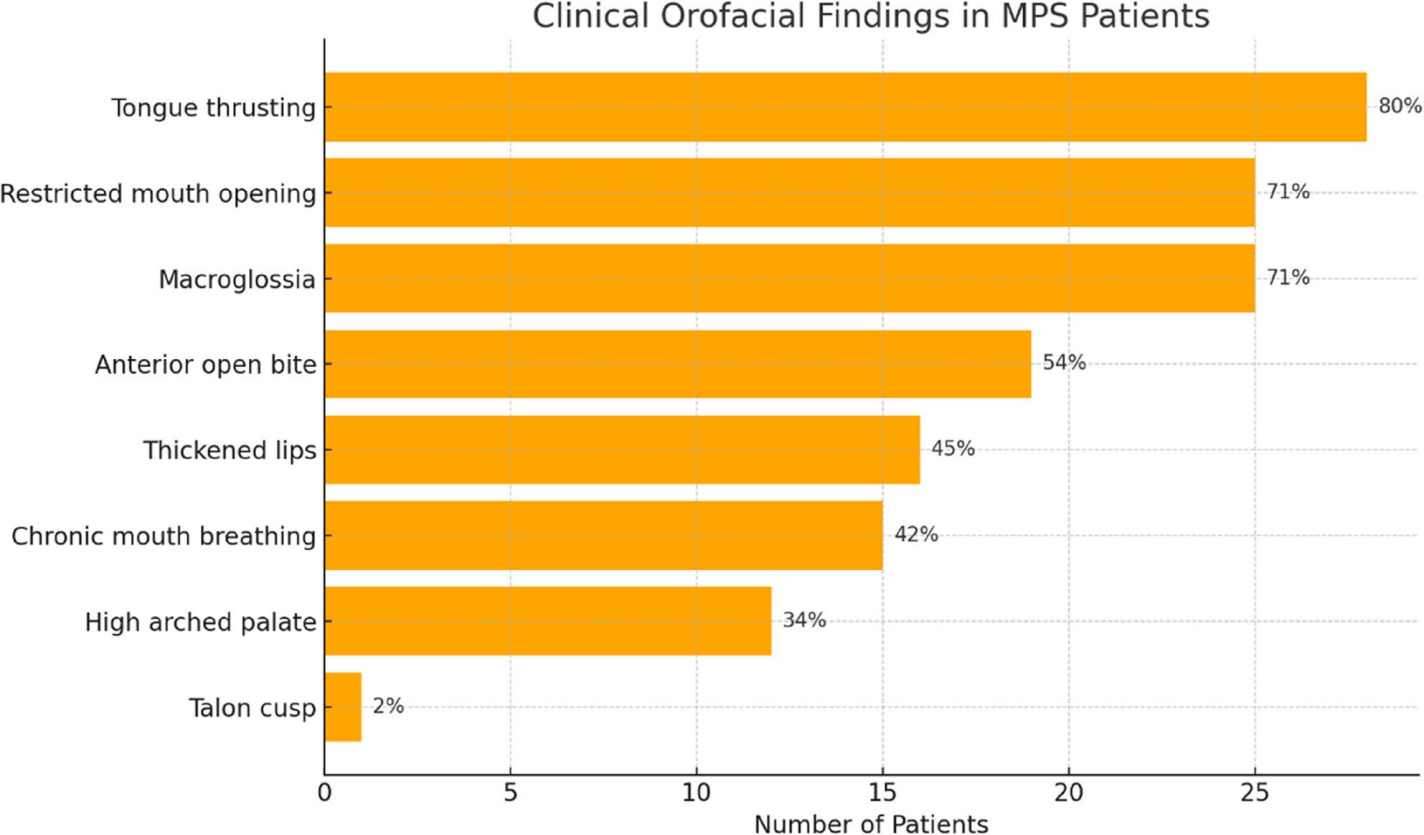
The clinical orofacial findings in MPS patients (*n* = 35)

**Fig. 3:**
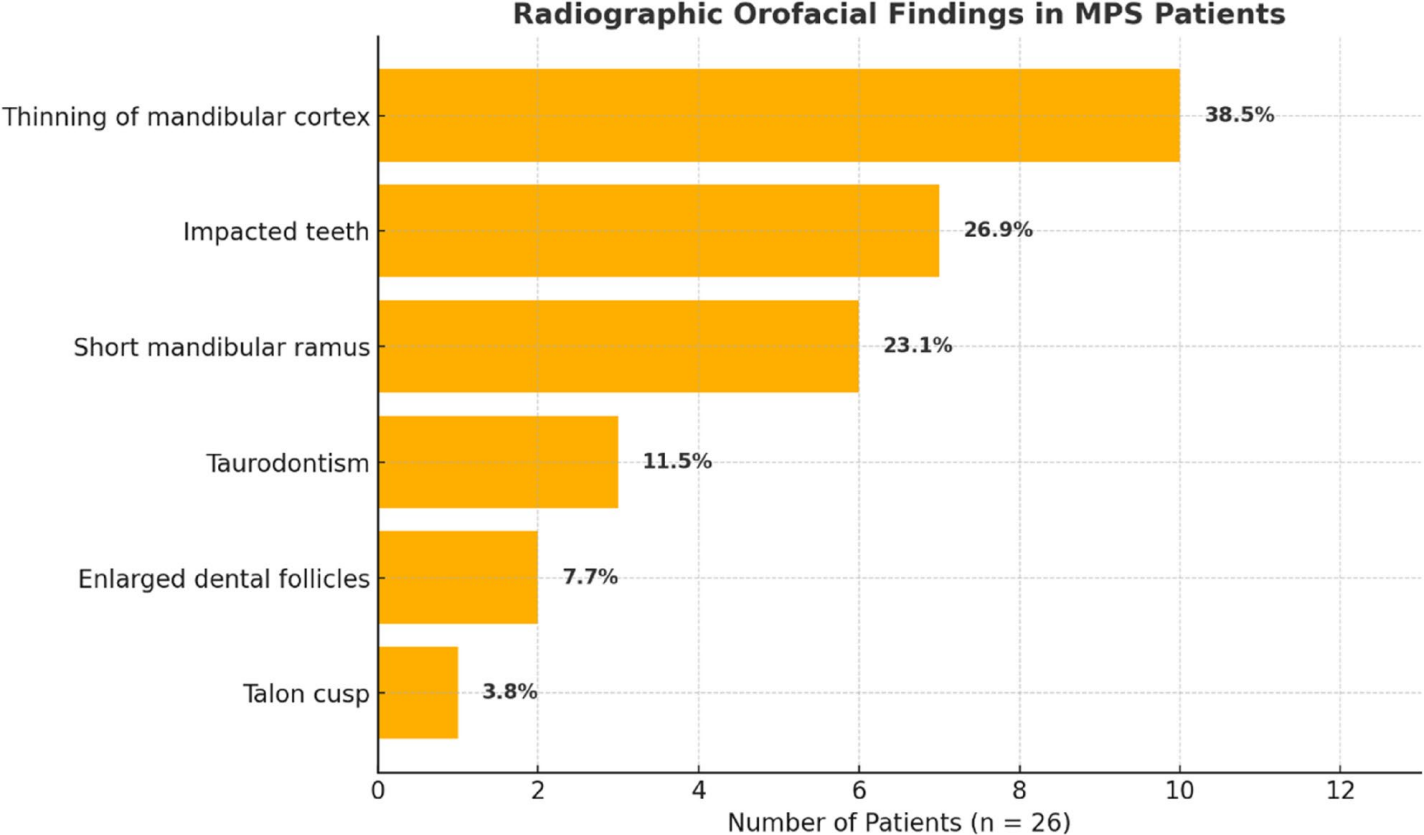
The radiographic orofacial findings in MPS patients (*n* = 26)

**Fig. 4:**
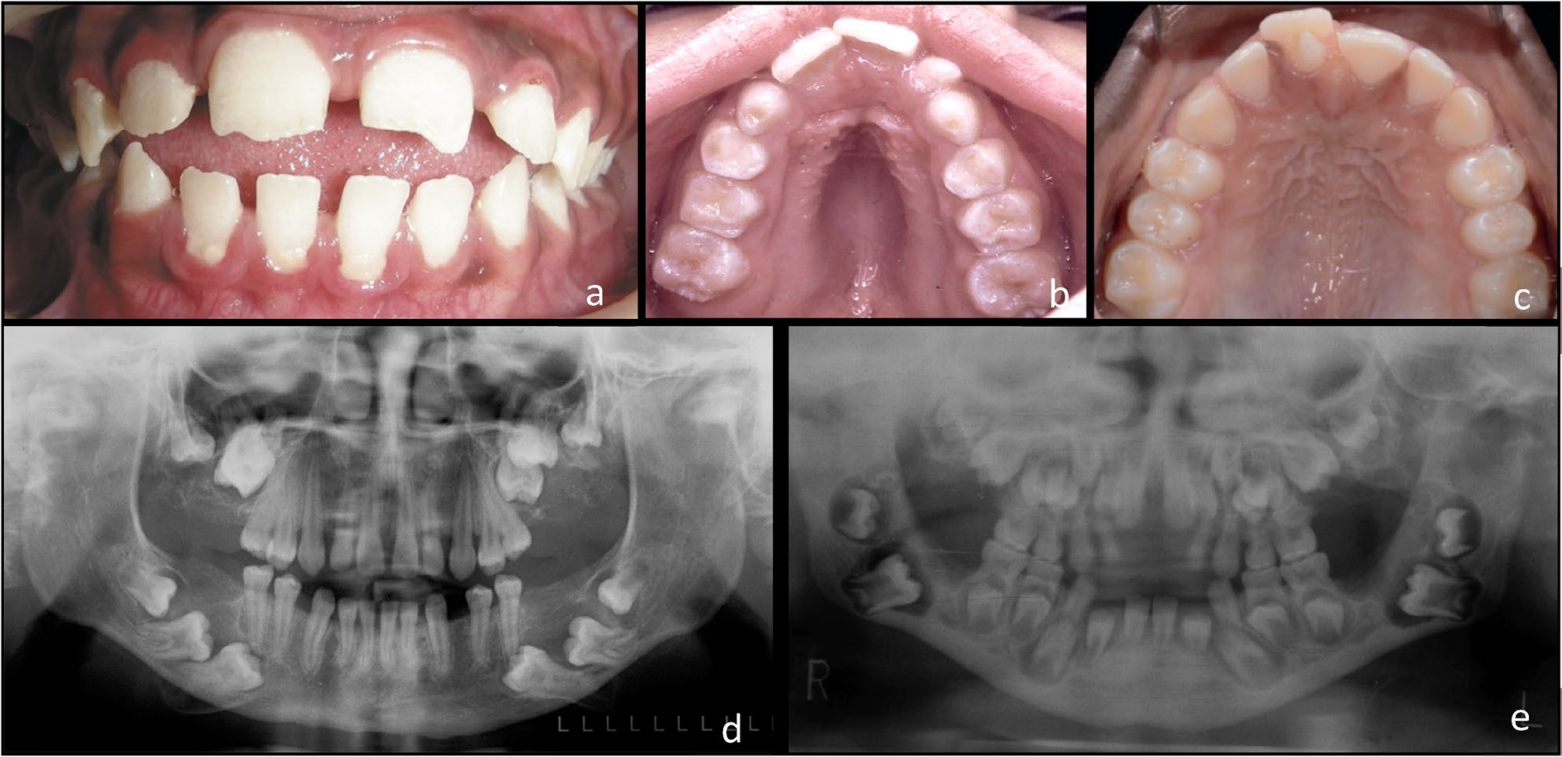
Representative clinical and panoramic radiographic findings in patients with mucopolysaccharidoses. (**a)** anterior open bite, (**b**) high arched palate, (**c**) talon cusp (**d**) thinning of mandibulaer cortex, impacted teeth, (**e**) short mandibular ramus, enlarged dental follicles. All images are anonymized and presented with informed consent

MPS are categorized into multiple clinically distinct types (I, II, III, IV, VI, VII and IX), each of which is associated with enzyme deficiencies and inheritance patterns [4, 5]. The overall estimated incidence of MPS ranges from 1.9 to 4.5 per 100,000 live births, although considerable geographical variation is observed in these figures [1–5]. In countries such as Türkiye, it is hypothesized that the prevalence is higher, primarily due to increased consanguinity, which has been demonstrated to increase the expression of autosomal recessive disorders [6].

In addition to the systemic and life-threatening manifestations, MPS frequently affects the craniofacial complex. Orofacial findings have been documented as some of the earliest and most consistent clinical features, frequently resulting in initial referral to dental specialists. The clinical features that are most frequently reported include macroglossia, thickened lips, gingival hyperplasia, anterior open bite, delayed eruptions and malocclusion. These findings are the result of both soft tissue infiltration and skeletal abnormalities. These orofacial features have the capacity to exert a substantial influence on such functions as mastication, verbal communication, airway patency and the general quality of life [7–10].

Radiographic findings provide further evidence to support the presence of skeletal dysplasia in the maxillofacial region. These may include taurodontism, condylar hypoplasia, short mandibular rami, impacted teeth, and altered trabecular bone patterns resembling osteoporotic changes [11–13]. Despite the prevalence of such findings, the extant literature consists primarily of isolated case reports or limited case series, often focusing on individual MPS subtypes or narrowly defined clinical parameters.

Previous studies have reported a spectrum of dental anomalies in patients with MPS, including delayed tooth eruption, enamel hypoplasia, taurodontism, dental impactions, and occlusal abnormalities such as anterior open bite and macroglossia [14–16]. These orodental findings not only serve as early clinical indicators of the underlying metabolic disorder but also have significant implications for nutrition, speech development, and oral health–related quality of life. Furthermore, the frequent need for dental interventions under sedation or general anesthesia in this medically complex population underscores the importance of early diagnosis and preventive care [7, 8]. Despite these reports, most studies have been limited by small sample sizes or a narrow focus on specific MPS subtypes, highlighting the need for more comprehensive evaluations such as the present study.

The present study aims to address this gap by means of a systematic evaluation of the orofacial and radiographic features of a cohort of patients with different MPS subtypes. The objective of this study is to characterize the prevalence and distribution of clinical and radiographic findings to enhance the comprehension of dental manifestations in MPS. This will also highlight the pivotal role of dental professionals in the early detection and multidisciplinary care of patients.

## Materials and methods

### Study design and ethical considerations

This observational, cross-sectional study was conducted in accordance with the ethical principles outlined in the Declaration of Helsinki and received approval from the Ethics Committee of Ege University, Faculty of Medicine (Reference number:15 − 10/7). Written informed consent was obtained from the paticipants and their parents prior to inclusion in the study.

### Study population

The study population consisted of 35 pediatric patients diagnosed with mucopolysaccharidosis (MPS), including subtypes I, II, III, IV, VI and VII. The diagnosis was confirmed by enzymatic and/or genetic testing performed by a certified metabolic disease center. All participants were referred to the Department of Pediatric Dentistry for a comprehensive oral and dental assessment as part of their routine multidisciplinary care.

### Inclusion criteria

- Confirmed diagnosis of MPS by clinical, biochemical or molecular methods.
- Age between 4 and 18 years.
- Ability to cooperate with intraoral examination.

### Exclusion criteria

- Lack of parental consent.
- Medical instability or acute systemic decompensation.
- Uncooperative behaviour or intellectual disability in clinical examination.
- A radiographic examination (panoramic imaging) was performed in all patients who were able to tolerate the procedure without distress, yielding evaluable images in 26 of the 35 cases.

### Clinical examination protocol

All clinical examinations were conducted by two pediatric dentists (D.Ç., E.K.A.) with extensive experience in managing medically complex and syndromic patients. We acknowledge that clinical assessment in children with mucopolysaccharidosis can be inherently challenging due to limited cooperation, anatomical constraints, and systemic comorbidities; however, the involvement of highly experienced pediatric dentists ensured methodological consistency, accurate detection of anomalies, and optimal patient comfort. Prior to data collection, both examiners participated in structured training sessions on standardized diagnostic criteria for orofacial anomalies in MPS patients. Calibration exercises were performed using a set of representative clinical photographs and panoramic radiographs from non-study cases. Each examiner independently evaluated identical cases, and inter-examiner reliability was calculated using Cohen’s kappa statistics, achieving a kappa value > 0.85, indicating excellent agreement. Any discrepancies were resolved through consensus discussions prior to the commencement of patient recruitment.

The decayed, missing, and filled teeth (DMFT/dft), decayed, missing, and filled surfaces (DMFS/dfs) indices were recorded according to WHO criteria [17]. The dental plaque scores were determined according to Silness & Löe index [18]. Each child underwent a detailed orofacial examination which included the following parameters: macroglossia, tongue thrust, limitation of mouth opening, anterior open bite, facial symmetry and soft tissue profile, palatal morphology, lip thickness and tone.

To ensure diagnostic consistency and minimize inter-examiner variability, objective criteria were applied for the evaluation of macroglossia and limited mouth opening. Macroglossia was defined as a tongue width exceeding the mandibular intercanine distance at rest or protrusion beyond the occlusal plane, following the criteria proposed by [19]. Limited mouth opening was assessed using the maximum interincisal distance, with values < 40 mm in children older than 6 years considered abnormal, in accordance with pediatric oral health guidelines [20].

Clinical findings were recorded using a structured case report form. Photographic documentation was undertaken with parental consent to aid qualitative analysis and record keeping.

Panoramic radiographs were taken using a standardized protocol on a digital panoramic unit. Radiographic analysis was performed independently by experienced pediatric dentists. Panoramic radiographs were considered for all syndromic patients, irrespective of the presence or absence of clinically evident dental anomalies. This approach served two principal purposes: (1) to generate comprehensive, high-quality data for the scientific literature, thereby enabling the identification and documentation of additional or previously unreported craniofacial manifestations such as the novel finding of a talon cusp in MPS-IV in the present study and (2) to facilitate the early detection and timely management of subclinical dental or skeletal anomalies before disease progression. All radiographic examinations were performed in strict accordance with the ALADAIP principle (As Low As Diagnostically Acceptable, being Indication-oriented and Patient-specific), ensuring that imaging was clinically justified, diagnostically adequate, and tailored to the individual needs of each patient, while adhering to standardized pediatric radiology protocols for anatomical assessment and clinical decision-making. The following radiographic variables were assessed as mandibular cortical thickness, ramus height and morphology, condylar and coronoid morphology, presence of impacted or ectopic teeth, developmental anomalies such as taurodontism, talon cusp, and enlarged dental follicles, trabecular bone pattern, mandibular notch flatness and condylar defect.

Radiographs were excluded from analysis of insufficient quality or if behavioural limitations prevented image acquisition.

### Statistical analysis

Data was analyzed using IBM SPSS Statistics for Windows, version 25.0 (IBM Corp., Armonk, NY, USA). Descriptive statistics were employed to summarize both categorical and continuous variables. Frequencies and percentages were reported for categorical variables, while medians and interquartile ranges were calculated for continuous data, as most variables did not follow a normal distribution.

Orofacial clinical and radiographic findings were stratified by MPS subtype (MPS-I, II, III, IV, VI, and VII) to identify potential subtype-specific patterns. Given the relatively small and uneven distribution of cases across subtypes, inferential analysis was necessarily conservative. Fisher’s Exact test was used in place of the chi-square test when expected cell counts were < 5, which applied to the majority of comparisons between subgroups. A two-tailed p-value of < 0.05 was considered statistically significant.

## Results

### Patient demographics and distribution of MPS subtypes

The study cohort consisted of 35 pediatric patients diagnosed with mucopolysaccharidosis (MPS), including 18 males (51.4%) and 17 females (48.6%), ranging in age from 4 to 18 years (mean age: 9.8 ± 3.7 years).

The distribution of MPS subtypes among the participants was given in Fig. 1.

The clinical examination was performed for all patients, although radiographs could not be obtained in 9 individuals due to severe cognitive limitations or behavioural non-cooperation.

### Clinical and radiographic characteristics of the study population

Clinical examination revealed multiple orofacial abnormalities consistent with the systemic involvement characteristic of mucopolysaccharidosis (MPS). Clinically, 24/35 (68.5%) patients had dental caries and 29/35 (83%) had dental plaque. The dental caries and dental plaque scores of the patients were given in Table 1. The most observed clinical features such as tongue thrusting, restricted mouth opening, macroglossia, and anterior open bite are summarized in Fig. 2. These findings were recorded across multiple MPS subtypes, with varying frequencies. Descriptive statistics were used to calculate the prevalence of each clinical manifestation. Comparative analysis between MPS subtypes was conducted using the chi-square test or Fisher’s Exact test with a significance threshold set at *p* < 0.05. Although no statistically significant differences were observed between subtypes for most clinical features, macroglossia and restricted mouth opening were notably more prevalent among patients with MPS-I and MPS-VI. For instance, macroglossia was significantly more frequent among patients with MPS-I and MPS-VI compared to other subtypes (*p* = 0.041), and restricted mouth opening was found to be significantly associated with MPS-VI (*p* = 0.038). Fisher’s Exact test was selected for these analyses due to the categorical nature of the variables and the limited sample sizes per group.

**Table 1:** Dental caries and dental plaque scores of MPS patients (*n* = 35)

	Mean ± SD
DMFT	1.34 **±** 2.08
DMFS	2.29 **±** 3.83
dmft	2.91 **±** 3.77
dmfs	6.74 **±** 10.49
Dental plaque score	2.94 **±** 1.26

Radiographic evaluation, based on panoramic radiographs, was successfully performed in 26 of the 35 patients (74.3%). The main radiological findings are summarized in Fig. 3, including thinning of the mandibular cortex (38.5%), impacted teeth (26.9%), and short mandibular ramus (23.1%). These features were more frequently observed in patients with MPS-IV and MPS-VI, although the small sample size limited the statistical power for subgroup analysis. No statistically significant associations were observed between subtype and abnormalities such as impacted teeth (*p* = 0.071). Taurodontism, enlarged dental follicles, and altered trabecular bone patterns were observed sporadically. Notably, a talon cusp, an uncommon dental anomaly was incidentally identified on a maxillary permanent right central incisor in a patient with MPS-IV.

Representative clinical and radiographic findings from the cases included in the study are presented in Fig. 4.

## Discussion

This study confirms the high prevalence and clinical significance of orofacial manifestations in pediatric patients diagnosed with mucopolysaccharidosis (MPS), a finding consistent with the systemic and progressive nature of these lysosomal storage disorders. Among the most common features observed in our cohort were macroglossia, anterior open bite, tongue thrust and restricted mouth opening, all of which are thought to result from the progressive accumulation of glycosaminoglycans in soft tissues, connective structures and the craniofacial skeleton. These findings are consistent with the pathophysiological basis of MPS, in which defective enzymatic degradation of glycosaminoglycans leads to progressive tissue infiltration, fibrosis and architectural distortion.

The prevalence of anterior open bite (54%) and macroglossia (71%) in our cohort is consistent with previous studies. Ballıkaya et al. [14]. reported anterior open bite in almost half of their MPS patients, and highlighted gingival inflammation and radiographic mandibular abnormalities as additional common findings. Similarly, studies by Antunes et al. [15] and Kantaputra et al. [16] highlighted anterior open bite, high palate and thick lips as key clinical indicators in both MPS-IV and MPS-VI populations. The reproducibility of these findings among geographically and genetically diverse populations highlights the diagnostic relevance of orofacial manifestations as early clinical indicators of mucopolysaccharidosis, thereby emphasising their value in facilitating timely recognition and intervention. Previous literature has consistently demonstrated the diagnostic value of orofacial manifestations in MPS, particularly in the context of early recognition and multidisciplinary management [14–16]. These findings are not only important for diagnostic purposes but also have significant implications for airway management, nutrition, and speech development. As such, timely identification of these orodental features by dental professionals can facilitate earlier referral to metabolic specialists and prompt initiation of enzyme replacement therapy or other disease-modifying interventions.

Radiographic assessment remains an important part of the evaluation of MPS, particularly in cases where clinical examination is limited by mental retardation or difficulty in cooperation. Radiographic analysis was feasible in 26 patients, with 9 excluded due to severe intellectual disability in the present study. The panoramic radiographs revealed a thin mandibular cortex in 38% of patients evaluated, impacted teeth in 27%, and a short mandibular ramus in 23%. These skeletal abnormalities particularly those involving the condylar process and ramus height are known to contribute to facial dysmorphism, malocclusion, and functional impairment in mastication and articulation. Similar radiological features have been reported in literature. For example, Cavaleiro et al. [21] reported mandibular hypoplasia, taurodontism, and trabecular bone changes in over 50% of their MPS cohort, highlighting the value of radiological monitoring in dental and orthodontic planning. Radiographic evaluations have further confirmed the skeletal involvement of the maxillofacial complex in MPS. Reports by Schmid-Herrmann et al. [22] and Lachman et al. [23] have detailed alterations in mandibular morphology, including reduced ramus height, condylar dysplasia, taurodontism, and changes in trabecular bone density. These radiological features, while often asymptomatic in early stages, may progress to cause significant malocclusion, facial asymmetry, and temporomandibular dysfunction if not addressed through coordinated dental and orthodontic care. Consequently, panoramic imaging serves not only as a diagnostic adjunct but also as a tool for treatment planning and longitudinal monitoring of disease progression.

A particularly novel finding of the present study was the identification of a talon cusp in a patient with MPS-IV an anomaly that, to our knowledge, has not been previously described in the context of MPS. Although talon cusp is relatively rare developmental dental anomaly, its presence in MPS patients may reflect aberrant odontogenesis associated with the broader metabolic disorder characteristic of the disease. This observation suggests that dental anomalies in MPS may be more diverse than previously appreciated and highlights the importance of comprehensive phenotypic documentation.

The frequent necessity for dental treatment under sedation or general anesthesia in this population is complicated by the multisystemic involvement of MPS, particularly airway compromise and cardiopulmonary comorbidities. Consequently, the role of pediatric dentists extends beyond the provision of diagnoses to encompass preventive care, treatment planning, and interdisciplinary coordination [24, 25].

It is important to note that dental professionals are frequently among the first healthcare providers to encounter the orofacial manifestations of systemic diseases. It can thus be concluded that increasing awareness of the oral phenotype associated with MPS may contribute to earlier referral, diagnosis, and initiation of disease-specific therapies such as enzyme replacement therapy or haematopoietic stem cell transplantation. These can mitigate systemic progression if instituted early [26, 27].

The findings of this study emphasize the necessity for integrating dental evaluations into the routine multidisciplinary care of MPS patients. Collaboration between pediatric dentists, pediatricians, metabolic specialists, radiologists, anesthesiologists and other allied professionals is essential to ensure comprehensive, safe and effective management that is tailored to the complex needs of this medically vulnerable population.

It is important to emphasize that radiological diagnostics are not inherently more complex than clinical assessments in children with MPS; in fact, panoramic imaging often provides superior visualization of skeletal and developmental anomalies compared to clinical examination alone. However, in this medically complex population, practical barriers such as limited behavioral cooperation, airway restrictions, and systemic comorbidities may hinder the acquisition of radiographs. These constraints explain why imaging could not be performed in all patients in the present study, rather than any intrinsic difficulty in radiological interpretation itself.

This study presents a detailed clinical and radiographic characterization of orofacial findings in pediatric patients with MPS, representing one of the most extensive single-center cohorts reported to date. The implementation of a standardized examination protocol, combined with objective radiographic assessment and the application of appropriate statistical methodologies contributes to methodological robustness and enhances the reliability and interpretability of the findings. A particularly noteworthy observation is the incidental identification of a talon cusp in a patient with MPS-IV, a finding that, to the authors’ knowledge, has not been previously documented in the context of MPS, thereby constituting a novel contribution to the existing literature. Nonetheless, several limitations should be acknowledged. The relatively small overall sample size, along with the unequal distribution of patients among MPS subtypes, constrained the statistical power of intergroup comparisons. Additionally, radiographic imaging could not be performed in a subset of patients due to cognitive or behavioural challenges, which may have introduced selection bias into the imaging analysis. The cross-sectional nature of the study also precludes the assessment of temporal changes or treatment-related effects. Finally, the inclusion of objective criteria for macroglossia and limited mouth opening in this study mitigates examiner-dependent variability, thereby enhancing reproducibility and comparability with future research.

## Conclusion

Orofacial manifestations are prevalent and clinically significant in mucopolysaccharidosis, frequently providing early diagnostic indications. Pediatric dentists play a vital role in recognizing these features, with radiographic evaluation serving as a valuable diagnostic adjunct. The integration of dental assessments into multidisciplinary care has been demonstrated to facilitate early diagnosis, guide management, and improve quality of life in affected children.

## Data Availability

The datasets used and/or analyzed during the current study are available from the corresponding author on reasonable request.

## References

[1] de Santana Sarmento DJ, de Carvalho SH, Melo SL, Fonseca FR, Diniz DN, Bento PM, et al. Mucopolysaccharidosis: radiographic findings in a series of 16 cases. Oral Surg Oral Med Oral Pathol Oral Radiol. 2015;120(6):e240-6. 26455293 10.1016/j.oooo.2015.08.009

[2] Fonseca FR, de Santana Sarmento DJ, Vasconcelos Medeiros PF, Diniz DN, dos Santos MT. Patients with mucopolysaccharidosis have tendencies towards vertical facial growth. J Oral Maxillofac Surg. 2014;72(12):2539–46. 25262398 10.1016/j.joms.2014.07.006

[3] Baehner F, Schmiedeskamp C, Krummenauer F, et al. Cumulative incidence rates of the mucopolysaccharidoses in Germany. J Inherit Metab Dis. 2005;28:1011–7. 16435194 10.1007/s10545-005-0112-z

[4] Achiatar LS, Hazoor HB, Adwani R, Patel VK, Gul A. Challenges in diagnosing and managing Hurler syndrome: a case report. Cureus. 2024;16(8):e67056. 39286678 10.7759/cureus.67056 PMC11405063

[5] Meikle PJ, Hopwood JJ, Clague AE, Carey WF. Prevalence of lysosomal storage disorders. JAMA. 1999;281:249–54. 9918480 10.1001/jama.281.3.249

[6] Koc I. Prevalence and sociodemographic correlates of consanguineous marriages in Turkey. J Biosoc Sci. 2008;40:137–48. 18028574 10.1017/S002193200700226X

[7] Ribeiro EM, Fonteles CS, Freitas AB, da Silva Alves KS, Monteiro AJ, da Silva CA. A clinical multicenter study of orofacial features in 26 Brazilian patients with different types of mucopolysaccharidosis. Cleft Palate Craniofac J. 2015;52(3):352–8. 24919127 10.1597/13-204

[8] Alpöz AR, Coker M, Celen E, Ersin NK, Gökçen D, van Diggelenc OP, Huijmansc JG. The oral manifestations of Maroteaux-Lamy syndrome (mucopolysaccharidosis VI): a case report. Oral Surg Oral Med Oral Pathol Oral Radiol Endod. 2006;101(5):632–7. 16632276 10.1016/j.tripleo.2005.06.023

[9] Turra GS, Schwartz IV. Evaluation of orofacial motricity in patients with mucopolysaccharidosis: a cross-sectional study. J Pediatr (Rio J). 2009;85(3):254–60. 19492172 10.2223/JPED.1899

[10] de Almeida-Barros RQ, de Medeiros PFV, de Almeida Azevedo MQ, de Oliveira Lira Ortega A, Yamamoto ATA, Dornelas SKL, Bento PM. Evaluation of oral manifestations of patients with mucopolysaccharidosis IV and VI: clinical and imaging study. Clin Oral Investig. 2018;22(1):201–8. 28315965 10.1007/s00784-017-2100-8

[11] Sarmento DJS, de Araújo TK, Mesquita GQTB, Diniz DN, Alves Fonseca FR, Medeiros PFV, Santos MTBRD, Godoy GP. Relationship between occlusal features and enzyme replacement therapy in patients with mucopolysaccharidoses. J Oral Maxillofac Surg. 2018;76(4):785–92. 29102600 10.1016/j.joms.2017.10.003

[12] Carneiro NCR, Abreu LG, Milagres RMC, Amaral TMP, Flores-Mir C, Pordeus IA, et al. Dental and maxillomandibular incidental findings in panoramic radiography among individuals with mucopolysaccharidosis: a cross-sectional study. J Appl Oral Sci. 2021;29:e20200978. 33886944 10.1590/1678-7757-2020-0978 PMC8054648

[13] Muthukumar Ramalingam S, Srinivasan D, ArunKumar S, ChiriyanKandath JL, Kaliamoorthy S. Morquio’s syndrome: a case report of two siblings. Case Rep Dent. 2017;2017:6176372. 28191355 10.1155/2017/6176372 PMC5278205

[14] Ballıkaya E, Eymirli PS, Yıldız Y, Avcu N, Sivri HS, Uzamış-Tekçiçek M. Oral health status in patients with mucopolysaccharidoses. Turk J Pediatr. 2018;60(4):400–6. 30859764 10.24953/turkjped.2018.04.007

[15] Antunes LA, Nogueira AP, Castro GF, Ribeiro MG, de Souza IP. Dental findings and oral health status in patients with mucopolysaccharidosis: a case series. Acta Odontol Scand. 2013;71(1):157–67. 22376155 10.3109/00016357.2011.654255

[16] Kantaputra PN, Kayserili H, Güven Y, Kantaputra W, Balci MC, Tanpaiboon P, et al. Oral manifestations of 17 patients affected with mucopolysaccharidosis type VI. J Inherit Metab Dis. 2014;37(2):263–8. 23974652 10.1007/s10545-013-9645-8

[17] World Health Organization. Oral health surveys: basic methods, 5th edn. 2013.

[18] Silness J, Löe H. Periodontal disease in pregnancy part II. Correlation between oral hygiene and periodontal condition. Acta Odontol Scand. 1964;22:121–35. 14158464 10.3109/00016356408993968

[19] Maher JL, Mahabir RC, Read LA. Acute macroglossia in the pediatric patient: worth a look. Pediatr Emerg Care. 2011;27(10):948–9. 21975495 10.1097/PEC.0b013e3182309c43

[20] Kumari S, Kumari S, Kumari A. The normal range of maximal incisal opening in pediatric subjects. J Pediatr Dent Oral Health. 2019;1(2):1–4.

[21] Cavaleiro RM, Pinheiro Md, Pinheiro LR, Tuji FM, Feio Pdo S, de Souza IC, Feio RH, de Almeida SC, Schwartz IV, Giugliani R, Pinheiro JJ, Santana-da-Silva LC. Dentomaxillofacial manifestations of mucopolysaccharidosis VI: clinical and imaging findings from two cases, with an emphasis on the temporomandibular joint. Oral Surg Oral Med Oral Pathol Oral Radiol. 2013;116(2):e141–8. 23849382 10.1016/j.oooo.2013.04.021

[22] Schmid-Herrmann CU, Muschol NM, Fuhrmann VU, Koehn AF, Lezius S, Kahl-Nieke B, et al. Mandibular condyle morphology among patients with mucopolysaccharidosis: an observational study of panoramic radiographs. Int J Paediatr Dent. 2022;32(5):737–44. 34967064 10.1111/ipd.12952

[23] Lachman RS, Burton BK, Clarke LA, Hoffinger S, Ikegawa S, Jin DK, et al. Mucopolysaccharidosis IVA (Morquio A syndrome) and VI (Maroteaux-Lamy syndrome): under-recognized and challenging to diagnose. Skeletal Radiol. 2014;43(3):359–69. 24389823 10.1007/s00256-013-1797-y PMC3901942

[24] Al Malak A, Issawi H, Hassoun M, Al Halabi M. Pediatric interventions in a Sanfilippo syndrome patient under general anesthesia: a case report. Case Rep Dent. 2025:7892363. 10.1155/crid/7892363 PMC11729508 39811794

[25] Saha S, Priya K, Rai K, Shetty RM, Hegde KM, Rao A, Abhijit Tanna KA. D, S M, S S. Case report: holistic dental care for a child with Hunter syndrome: addressing dental ramifications, overcoming challenges, and enhancing quality of life. F1000Res. 2024;13:268. 10.12688/f1000research.146468.1 PMC11134137 38812528

[26] Nagpal R, Georgi G, Knauth S, Schmid-Herrmann C, Muschol N, Braulke T, et al. Early enzyme replacement therapy prevents dental and craniofacial abnormalities in a mouse model of mucopolysaccharidosis type VI. Front Physiol. 2022;13:998039. 36213247 10.3389/fphys.2022.998039 PMC9532570

[27] Clarke LA, Harmatz P, Fong EW. Implementing evidence-driven individualized treatment plans within Morquio A syndrome. Mol Genet Metab. 2016;117(2):217. 26877092 10.1016/j.ymgme.2015.12.157

